# MND1 regulates PANoptosis and stemness of lung adenocarcinoma cells by stabilizing RCOR2 mRNA

**DOI:** 10.1038/s41419-026-09012-5

**Published:** 2026-07-10

**Authors:** Lianlian Ouyang, Xingjie Tang, Shiyao Jiang, Yating Liu, Na Liu, Chao Mao, Yuanbing Chen, Ye Fan, Ziyun Zhang, Jingjing Huang, Yongguang Tao, Xing Yu, Li Cong, Yiqun Jiang

**Affiliations:** 1 School of Basic Medical Sciences, Hunan Normal University Health Science Center, Hunan Normal University, Changsha, Hunan China; 2 Department of Pathology, Xiangya Hospital, Central South University, Changsha, Hunan China; 3 Department of Pharmacy, The Third Xiangya Hospital, Central South University, Changsha, Hunan China; 4 School of Basic Medicine, Central South University, Changsha, Hunan China; 5 Department of Pathology, Nanjing Drum Tower Hospital, State Key Laboratory of Pharmaceutical Biotechnology, School of Life Sciences, Nanjing University, Nanjing, China; 6 Department of Neurosurgery, The Third Xiangya Hospital, Central South University, Changsha, Hunan China; 7 The Second People’s Hospital of Hunan Province/Brain Hospital of Hunan Province, Changsha, Hunan China; 8 Hunan Key Laboratory of Cancer Metabolism, Hunan Cancer Hospital and The Affiliated Cancer Hospital of Xiangya School of Medicine, Central South University, Changsha, Hunan China; 9 Key Laboratory of Model Animals and Stem Cell Biology in Hunan Province, Hunan Normal University School of Medicine; Engineering Research Center of Reproduction and Translational Medicine of Hunan Province; Manufacture-Based Learning and Research Demonstration Center for Human Reproductive Health New Technology of Hunan Normal University, Changsha, Hunan China; 10 School of Basic Medical Sciences, Hunan Normal University, Changsha, China; Biliary Tract Surgery Laboratory, Department of Hepatobiliary Surgery, Hunan Provincial People’s Hospital, The First Affiliated Hospital of Hunan Normal University, Changsha, Hunan China

**Keywords:** Non-small-cell lung cancer, Cancer stem cells

## Abstract

PANoptosis is an inflammatory programmed cell death mechanism that depends on the PANoptosome complex, which integrates hallmark features of apoptosis, necroptosis, and pyroptosis. Meiotic nuclear division protein 1 (MND1), a meiosis-specific protein, primarily participates in homologous chromosome pairing and DNA double-strand break repair. However, the precise role of MND1 in regulating PANoptosis and tumor stemness remains unclear. Here, we provide evidence that MND1 participates in the regulatory mechanisms of PANoptosis and cell stemness in lung adenocarcinoma (LUAD). Mechanistically, MND1 recruits PABPC1 to interact with the 3’-UTR of RCOR2 mRNA, significantly enhancing its stability. As a crucial component of the pluripotency regulatory network, the stable expression of RCOR2 is essential for maintaining tumor cell stemness. Our findings suggest MND1 acts as an oncogene in LUAD by stabilizing RCOR2 mRNA, maintaining the stemness of LUAD and inhibiting PANoptosis. Notably, this study represents the first investigation to elucidate the regulatory role of MND1 in modulating both stemness properties and PANoptosis in LUAD, providing valuable insights into the molecular mechanisms underlying LUAD pathogenesis.

## Introduction

Lung cancer represents the most prevalent and lethal malignant tumor worldwide in terms of both incidence and mortality, including non-small cell lung cancer (NSCLC) and small cell lung cancer (SCLC), of which NSCLC represents the predominant histological type, constituting approximately 80–85% of all cases [1]. NSCLC is further histologically classified into lung adenocarcinoma and lung squamous cell carcinoma, and more than half of the cases are LUAD [2]. Notwithstanding the advancements achieved in lung cancer treatment in recent years, many therapeutic approaches remain unsatisfactory. Multiple factors such as metastasis, recurrence, heterogeneity, chemoradiotherapy resistance, and immunosurveillance evasion lead to the failure of cancer treatment [3]. Notably, the presence of lung cancer stem cells (LCSCs) is a leading cause of therapeutic failure.

Among tumor cells, there exists a subset of cells with self-renewal and differentiation potential, and these cells with stem-like properties are considered as cancer stem cells (CSCs) [4]. Cell surface markers, comprising the stem cell-associated antigen CD133, the hyaluronan receptor CD44, the proliferation-associated glycoprotein CD166, the aldehyde dehydrogenase (ALDH), and the epithelial cell adhesion molecule (EpCAM), are currently used as alternative markers for prospectively identifying cell populations that fulfill the functional definition of CSCs [5]. Emerging as a research focus, LCSCs are pivotal in both the occurrence and progression of lung cancer. Evidence indicates that a rare subset of stem-like tumor cells within lung cancers possess high tumorigenic ability in vivo in lung cancer. These cells survive various therapies for lung cancer, such as radiotherapy, chemotherapy, targeted therapy, etc., driving recurrence and drug resistance [6]. Notably, despite comprising only a minor subset of primary tumor cells, stemness is not an intrinsic or immutable property but rather an acquired phenotype dynamically modulated by a multitude of factors, including transcription factors, signaling pathways, and the tumor microenvironment that collectively affect the proportion and function of tumor stem cells [7, 8]. Therefore, demonstrating the molecular mechanisms governing the initiation and maintenance of stemness properties in LUAD is crucial for improving current treatments and reducing tumor recurrence.

In response to alterations in the extracellular milieu, cells may undergo diverse modalities of programmed cell death, such as apoptosis, necrosis, and pyroptosis [9]. These programmed cell death (PCD) pathways are involved in many disease processes [10]. Impaired death pathway is an important characteristic of tumor cells and the primary contributor to chemoresistance and unfavorable patient outcomes [11]. While they differ in mechanisms, growing evidence has indicated a strong correlation among them [12]. Recent research has shown that the signaling pathways of pyroptosis, apoptosis, and necroptosis can be simultaneously activated within a single cell, giving rise to a novel cell death modality termed PANoptosis (an acronym derived from pyroptosis, apoptosis, and necroptosis) [10, 13]. PANoptosis has emerged as a research focus in cancer. Numerous chemotherapeutic agents are capable of concurrently initiating apoptosis, necrosis, and pyroptosis, thereby promoting cellular demise and inhibiting tumor proliferation. For instance, oxaliplatin, a first-line gastric cancer chemotherapeutic, has been proven to induce PANoptosis [14]. An expanding body of evidence has reported that PANoptosis is involved in diverse cancer-related processes, including chemoresistance in colorectal cancer and the immunotherapeutic response in gastric cancer [10, 15]. However, its role in lung adenocarcinoma remains underexplored.

MND1 is a protein exclusively present during meiosis, playing a crucial role in facilitating homologous chromosomal pairing and mediating DNA double-strand break repair [16]. Recent studies emphasize the potential significance of meiotic factors as valuable targets for tumor therapy and biomonitoring [17–19]. Notably, elevated expression of MND1 has been consistently associated with the pathogenesis and progression of various malignancies, including non-small cell lung cancer [20], hepatocellular carcinoma [21], and renal clear cell carcinoma [22]. Particularly, MND1 exhibits significantly elevated expression levels in lung cancer, suggesting that it may serve as a prognostic biomarker for unfavorable clinical outcomes [23]. Additionally, it has been demonstrated that MND1 collaborates with KLF6 and E2F1 to control the cell cycle, encouraging the growth of LUAD [20]. These studies indicated that MND1 is critically involved in the pathogenesis of lung cancer. Nevertheless, the relationship among MND1, the LUAD PANoptosome, and stemness remains poorly understood.

REST Corepressor 2 (RCOR2) is a transcriptional regulatory protein that functions as a repressor and exhibits predominant expression in embryonic stem cells (ESCs). It plays an essential role in the maintenance of cellular pluripotency within these pluripotent stem cell populations [24]. Studies have shown that RCOR2 can functionally compensate for SOX2 in the reprogramming of somatic cells to generate induced pluripotent stem cells (iPSCs) [25]. Furthermore, RCOR2 has been identified as a component of the protein complex assembled on the Nanog promoter in embryonic stem cells (ESCs), providing evidence that it plays a functional role within the pluripotency regulatory network [25]. In gliomas, RCOR2 can replace OLIG2 to reprogram differentiated glioblastoma (GBM) cells into a stem-like state characterized by enhanced self-renewal capacity and tumor proliferation [26]. The findings indicate that RCOR2 likely plays a pivotal role in tumor stem cell reprogramming.

RNA binding proteins (RBPs) are essential regulators of RNA metabolism, primarily orchestrating key processes such as RNA splicing, transport, translation, and localization [27]. Poly (A)-binding protein cytoplasmic 1 (PABPC1) is a crucial RBP that initiates protein translation and mRNA decay, serving as a central modulator of cytoplasmic mRNA localization [28]. Recent evidence suggests that PABPC1 participates in regulating the mRNA decay and translation, implying that the interaction of PABPC1 with the poly(A) tail [27]. Moreover, PABPC1 has been demonstrated to participate in early steps in regulating mRNA expression [29]. Therefore, PABPC1 may be involved in RNA metabolism in lung adenocarcinoma cells.

In this study, we have innovatively explored a previously unreported mechanism through which MND1 regulates the development of lung adenocarcinoma. Bioinformatics analysis indicated that MND1 is involved in regulating lung adenocarcinoma stemness and PANoptosis. Furthermore, we have confirmed that MND1 recruits PABPC1 to interact with the 3’-UTR region of RCOR2 mRNA, thereby promoting its stability. In vitro and in vivo experiments showed that the mechanism promotes the stemness phenotype of lung adenocarcinoma. Meanwhile, we have demonstrated that MND1 inhibits PANoptosis, reducing inflammatory programmed cell death in these cells, revealing the important function of MND1 as an oncogene in antagonizing the cell death pathway. This finding not only shed light on MND1’s role in lung adenocarcinoma stemness and PANoptosis regulation, but also clarified the diagnostic value of MND1 and RCOR2 as potential biomarkers. Targeting the MND1-PABPC1-RCOR2 axis may provide a potential solution to overcome the high invasiveness and therapeutic resistance of lung adenocarcinoma.

## Materials and methods

### Data collection

We collected the transcriptional data and corresponding clinical information of 500 LUAD patients from The Cancer Genome Atlas (TCGA) database, as well as single-cell sequencing data GSE119911 and GSE148071 from the GEO database.

### Variance analysis

The R software package ‘limma’ [30] was employed to identify the differentially expressed genes (DEGs) between the MND1 high-expression and low-expression groups in TCGA dataset, as well as between SPCA-1 cells with stable MND1 knockout and the corresponding control group, and screened the criteria are |logFC | ≥ 1 and *p* < 0.05. The R package ‘ggplot2’ [31] is employed to generate volcano plots for visualizing the results of differential analysis.

### KEGG and GSVA analysis

Exploring the biological functions and signaling pathways associated with MND1 through KEGG pathway enrichment analysis and gene set variation analysis (GSVA). KEGG analysis was performed using the R software package ‘clusterProfiler’, while GSVA was conducted with the R software package ‘GSVA’ [32].

### The mRNAsi and cytotrace score based on mRNA expression

The mRNA expression-based stemness index (mRNAsi) is a machine learning-derived methodology that quantitatively assesses tumor stemness by analyzing gene expression profiles related to stemness. The score spans a range of 0–1, with higher values indicating increased stemness characteristics. The mRNAsi scores of LUAD patients in TCGA were extracted using the method proposed by M. Malta et al. [33]. Cytotrace is a powerful computational framework for predicting differentiation status from single-cell sequencing data. The CytoTRACE scores for LUAD samples in the GSE119911 dataset were computed using the R package ‘CytoTRACE v0.3.3’ [34].

### WGCNA network

Weighted gene co-expression network analysis (WGCNA) [35] was employed to construct a scale-free co-expression network utilizing RNA-seq data from The Cancer Genome Atlas (TCGA). Hierarchical clustering analysis identified a total of 9 non-gray modules. Among these modules, the one demonstrating the most robust correlation with both MND1 and mRNAsi was selected for subsequent in-depth investigation.

### Tumorsphere formation

Sphere Formation ability is a method for the identification of cancer stem cells in vitro. It is measured by the self-renewal ability of a single cell in an appropriate conditioned medium, which is generally expressed as Sphere Formation Efficiency (SFE). These measurements were conducted as previously described [36]. The number of tumorspheres with a diameter exceeding 100 μm was quantified under the microscope for subsequent statistical analysis after 7 days.

### In vitro and in vivo limiting dilution analysis (LDA)

Inoculate cells (0, 20, 40, 60, 80, or 100 cells per well) into a specially designed 96 well plate in vitro and maintain culture in spheroid culture medium. After 7–14 days of cultivation, quantify the frequency of sphere units using ELDA software based on the number of positive wells (http://bioinf.wehi.edu.au/software/elda) [37].

For the in vivo limiting dilution assays, stable cell lines of Control or koMND1 SPCA-1 were used for controlled dilution experiments. The mice randomly assigned to experimental groups received an intraperitoneal injection with 10 mg/kg pentobarbital sodium for anesthesia. Subsequently, the isolated single-cell suspension was diluted using a limited dilution series (1 × 10^6^, 1 × 10^5^, and 1 × 10^4^ cells) and inoculated subcutaneously into 4-week-old female BALB/c nude mice. Each experimental group consisted of 5 mice (*n* = 5), with a total of 30 mice used in this study. Xenografted mice were sacrificed by CO_2_ inhalation either when tumor volume reached approximately 500 mm^3^ or after 42 days of observation to assess tumor formation and tumor growth curve [37]. All animal procedures were conducted in accordance with the guidelines of the Institution Animal Care and Use Committee guidelines and were approved by the Hunan Normal University.

### RNA immunoprecipitation (RIP) assay

Place the cells in RIP lysis buffer on ice for 30 min, then incubate the cell lysate with 5 µg of normal IgG antibody (#S0001, Affinity) or MND1 antibody (#bs-7799R, Bioss, America), PABPC1 antibody (#10970-1-AP, Proteintech, China), and magnetic protein A/G beads at 4 °C overnight. Wash the magnetic beads three times and subsequently incubated with proteinase K for 30 min at 55 °C. Co-precipitated RNAs were isolated using Trizol reagent (R401-01, Vazyme, China) and quantified by RT-qPCR [38].

### Transwell assays

The Transwell assay is a common in vitro method for assessing cell migration and invasion abilities. Cell behavior is quantified by counting the number of migrated or invaded cells on the bottom of the chamber or in the culture medium. These measurements were conducted as previously described [39]. Randomly select 3 fields of view, take photos under a microscope, and count the images using ImageJ software (National Institutes of Health).

### Cell counting Kit-8 (CCK-8)

CCK-8 assay is a widely used method for cell proliferation and cytotoxicity analysis. These measurements were conducted as previously described [39]. Statistical analyses were conducted utilizing GraphPad Prism 8.02.

### Colony-forming assay

The colony formation assay is an in vitro method for determining cell viability and proliferative potential. It focuses on determining whether individual cells can grow into colonies containing at least 50 cells, thereby evaluating the ability of each cell within a population to undergo sustained clonal expansion. These measurements were conducted as previously described [40]. Visualize and count clone numbers using a microscope and ImageJ software.

### Statistical analyses

All statistical analyses and graphical representations were conducted using R software or GraphPad Prism. Group comparisons were performed employing either the Student’s *t*-test or the Wilcoxon signed-rank test. Spearman’s rank correlation analysis was employed to assess the relationships between variables. Statistical significance was determined at a two-tailed.

The remaining details of the experimental protocol are provided in the Supplementary Materials.

## Results

### MND1 is highly associated with PANoptosis and the stemness of LUAD

**Fig. 1:**
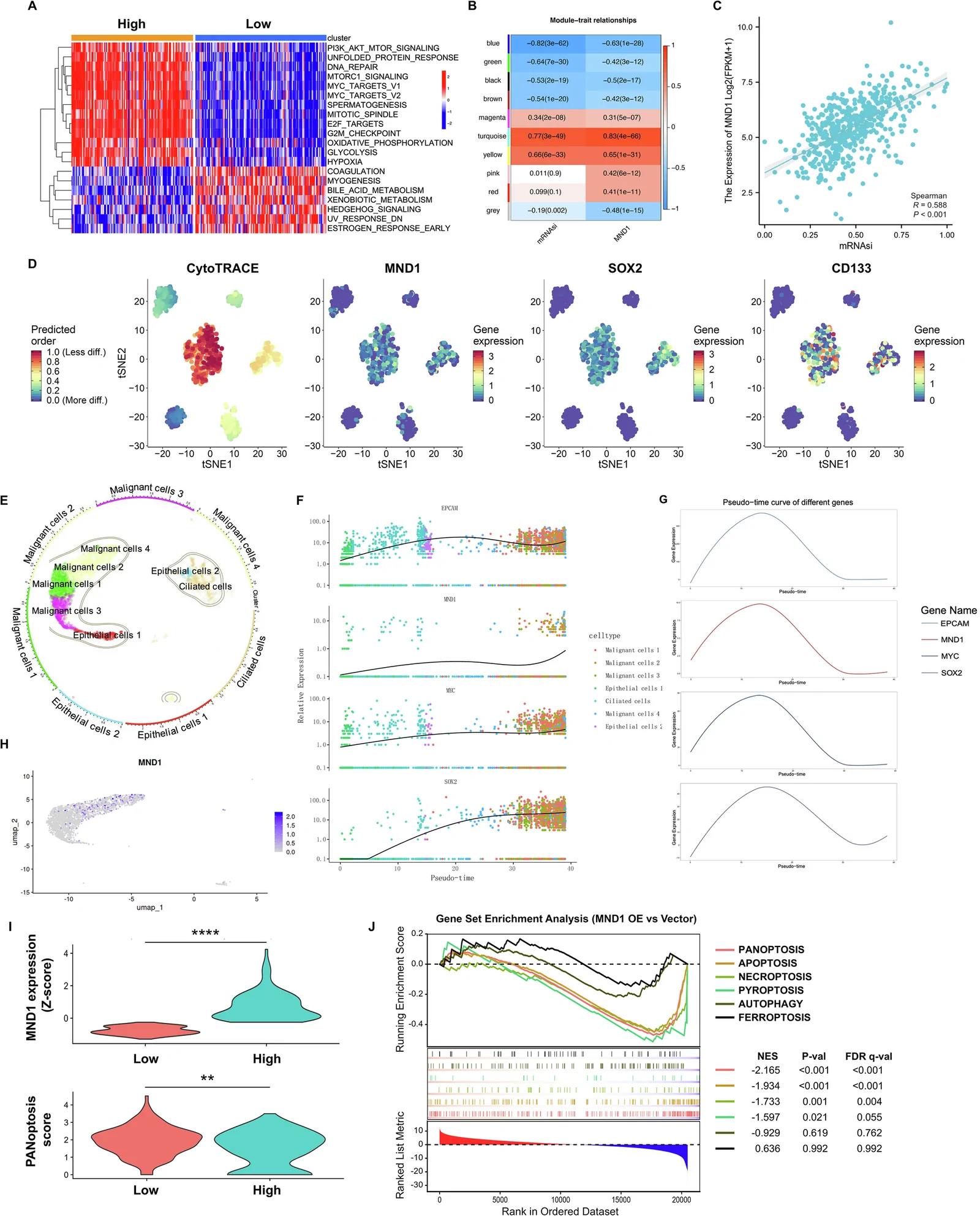
MND1 is highly associated with LUAD stemness. **A** GSVA of extreme values (high vs low) of MND1 from TCGA-LUAD (*n* = 250). **B** The heatmap depicts the correlation between various modules and mRNAsi and MND1. **C** The scatter plot reveals a significant correlation between MND1 and mRNAsi. **D** The t-SNE plot illustrates the distribution of CytoTRACE scores and MND1, SOX2, and CD133 expression in LUAD cells. **E** Selected genes within cancer cell subgroups exhibit expression changes across different cell types. **F** The differentiation changes within the selected cancer cell subgroups over pseudotime. **G** Selected genes within cancer cell subgroups exhibit expression changes over pseudotime. **H** UMAP showing the distribution of MND1 expression within the malignant cell cluster. **I** Violin plots of pan-apoptosis scores stratified by MND1 expression. **J** GSEA enrichment plots of regulated RCD gene sets based on DEGs after MND1 overexpression. Statistical analyses were performed using Prism GraphPad Software and tested by Pearson’s correlation analysis.

To comprehensively explore the biological significance of *MND1* in LUAD, a dataset comprising 500 LUAD samples from TCGA was stratified into four distinct subsets based on *MND1* mRNA expression levels utilizing the quartile method. A subset consisting of 250 samples, representing the extremes of *MND1* expression (high vs. low), was selected for detailed examination. GSVA heatmap analysis unveiled a pronounced enrichment of stemness-related pathways, including mTORC1 and MYC pathways [41], within the *MND1* high-expression cohort (Fig. 1A). Differential expression analysis conducted on the 250 samples (*MND1* high vs. low) identified 1596 upregulated genes and 2868 downregulated genes (Fig. S1A). These differentially expressed genes were subjected to WGCNA analysis, with a soft threshold (*β* = 4) selected optimal for constructing a co-expression network comprising 9 non-grey gene modules (Fig. S1B, C). The turquoise module was identified as the pivotal module, owing to its robust positive correlation with *MND1* (Cor = 0.83, *p* < 0.05) and the mRNAsi (Cor = 0.77, *p* < 0.05) (Fig. 1B). Notably, *MND1* was identified as a constituent of this module, exhibiting a significantly positive correlation with mRNAsi (R = 0.588, *p* < 0.001) (Fig. 1C).

To further confirm the correlation between MND1 and stemness, we calculated the CytoTRACE score [34] using the non-small cell lung cancer (NSCLC) single-cell sequencing data set GSE119911. In cell subpopulations with elevated Cytotrace scores, *MND1* exhibits high expression. Concurrently, stemness-related markers *SOX2* and *CD133* are abundant within these cell subpopulations (Fig. 1D). Utilizing the R Seurat package (version 5.1.0), we conducted quality control and cell clustering analyses on scRNA-seq datasets GSE119911 and GSE148071. Subsequently, integration anchors were employed to merge the two datasets, followed by downstream analyses (Fig. S1D). The resultant dataset comprised 6699 cells distributed across 19 clusters. Annotation of these 19 cell clusters was accomplished by referencing expression patterns of typical marker genes sourced from CellMarker 2.0 (Fig. 1E, Fig. S1D–F). To delineate distinct states of lung cancer cells, pseudotime trajectory analysis was conducted on seven selected cell clusters. Notably, the pseudotime progression depicted a transition of the lung epithelial cell population from a normal to a malignant state. Concurrently, the expression levels of *MND1* and stemness related genes such as *EPCAM, SOX2*, and *MYC* exhibited an ascending trajectory along the pseudotime axis (Fig. 1F, G, Fig. S1G–I). These findings indicate heightened *MND1* expression in LUAD cells characterized by elevated stemness. Furthermore, pseudo-temporal analysis of gene expression clustering heatmaps revealed consistent expression trends among *MND1, MYC*, and *SOX2* (Fig. S1J).

The “antagonistic balance” between tumor cell death and stemness constitutes a core regulatory axis underlying tumorigenesis, drug resistance, and immune escape [42, 43]. To further clarify the biological function of MND1, we analyzed the enrichment of regulated cell death (RCD)-related pathways in 500 LUAD samples. GSEA results showed significant enrichment in PANoptosis, apoptosis, pyroptosis, and necroptosis pathways, suggesting that MND1 might be involved in PANoptosis regulation (Fig. S1K). Correlation analysis confirmed that the expression of MND1 was negatively correlated with the expression of PANoptosis-related genes (Fig. S1L). Furthermore, we extracted malignant cells from the single-cell dataset and stratified them into high and low MND1 expression groups using the median MND1 expression as the cutoff (Fig. 1H). The PANoptosis score of each cell was calculated to evaluate its sensitivity to PANoptosis, and the results showed that the low MND1 expression group had a higher PANoptosis score and was more sensitive to PANoptosis (Fig. 1I). Similarly, we established MND1-overexpressing cell lines and performed RNA-sequencing (RNA-seq) analysis. Subsequent enrichment analysis of differentially expressed genes (DEGs) in RCD-related pathways revealed significant enrichment in PANoptosis, apoptosis, pyroptosis, and necroptosis pathways. Collectively, these indicate a significant association between MND1 and the stemness of LUAD as well as PANoptosis.

### MND1 Inhibits PANoptosis in Lung Adenocarcinoma

**Fig. 2:**
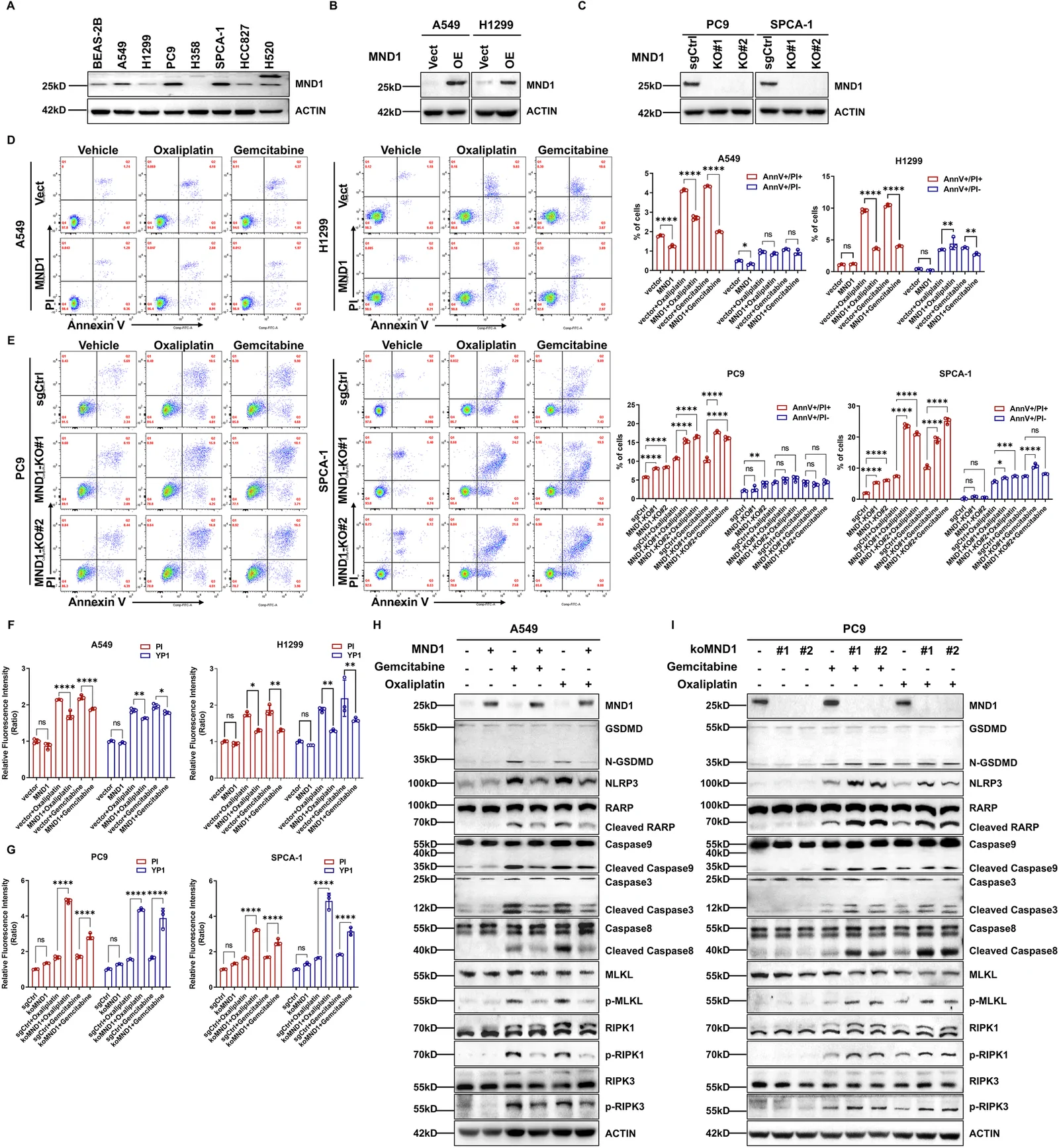
MND1 Inhibits PANoptosis in Lung Adenocarcinoma. **A** Western blot analysis was employed to assess the expression levels of the MND1 protein in LUAD cell lines. **B** Western blot analysis was used to verify the MND1 overexpression cell lines. **C** Western blot analysis was used to verify the MND1 knockdown cell lines. **D**, **E** Flow cytometry assays were used to examine the effect of MND1 on the apoptosis of cells treated with oxaliplatin and gemcitabine (10 μg/mL) for 24 h. **F**, **G** Treatment of control and LUAD cells overexpressing MND1 with 10 μg/ml of oxaliplatin and gemcitabine, respectively, for 24 hours. Positive YP1 (green) indicates necrotic apoptosis or apoptotic cells; positive PI (red) indicates necrotic apoptosis, apoptosis, or pyroptosis of cells. **H**, **I** Western blot analysis was employed to measure the expression levels of PANoptosis-related markers in both MND1-overexpressing and MND1-knockdown cells treated with oxaliplatin and gemcitabine for 24 h. The data in (**D**–**G**) represent three independent experiments and shown as the mean ± SD. Two-way ANOVA was performed with Tukey’s multiple comparisons test. ns nonsignificant (*P* > 0.05), *P < 0.05, **P < 0.01, ***P < 0.001, ****P < 0.0001.

To investigate the biological function of MND1 in LUAD, we examined the protein expression of MND1 in LUAD cell lines (Fig. 2A), and established MND1-overexpressing cell lines (A549 and H1299; Fig. 2B) as well as MND1-deficient cell lines (PC9 and SPCA-1; Fig. 2C). To further explore the relationship between MND1 and PANoptosis, we treated cells with oxaliplatin and gemcitabine. Oxaliplatin has been reported to induce PANoptosis in tumor cells [15], while gemcitabine is a first-line chemotherapeutic drug for lung cancer. Prior research has demonstrated that gemcitabine can induce apoptosis in tumor cells [44]. We hypothesize that MND1 exerts a functional role in mediating the response of LUAD cells to gemcitabine. We adopted the treatment concentrations of gemcitabine and oxaliplatin as reported in prior studies [45–48]. To evaluate the contribution of MND1 to gemcitabine sensitivity in LUAD cells, we performed comprehensive functional assays including flow cytometry and confocal laser microscopy. Notably, following MND1 overexpression, the rate of gemcitabine or oxaliplatin-induced apoptosis in A549 and H1299 cells was reduced (Fig. 2D), MND1 reduced the sensitivity of lung adenocarcinoma cells to death induction. Immunofluorescence results further indicated that overexpression of MND1 significantly reduced the cell death induced by gemcitabine or oxaliplatin (Fig. 2F, Fig. S2A, B). In contrast, ablation of MND1 in LUAD cells yielded contrasting outcomes. MND1 knockout combined with oxaliplatin or gemcitabine treatment significantly increased the proportion of early and late apoptotic cells, accompanied by a significant reduction in the number of viable cells (Fig. 2E). Immunofluorescence assays further confirmed a substantial elevation in apoptotic and necrotic cells under combined oxaliplatin or gemcitabine treatment after MND1 knockout. (Fig. 2G, Fig. S2C, D). The aforementioned findings indicate that MND1 inhibits the occurrence of PANoptosis in LUAD cells.

To further elucidate the molecular mechanisms through which MND1 regulates cell death pathways during treatment with gemcitabine and oxaliplatin, we systematically analyzed key downstream effectors. In the apoptosis pathway, we observed that the cleaved isoforms of caspase-3 and caspase-7 were significantly upregulated in MND1-silenced LUAD cells following gemcitabine or oxaliplatin treatment (Fig. 2I). Correspondingly, their upstream initiator caspases, caspase-8 and caspase-9 displayed a comparable cleavage activation pattern. Furthermore, silencing MND1 notably enhanced the chemotherapy-induced phosphorylation of MLKL, the essential necroptosis executioner protein. Correspondingly, we detected increased levels of phosphorylated RIPK1 and cleaved RIPK1, an upstream signaling molecule that mediates MLKL phosphorylation and facilitates crosstalk between apoptosis and necroptosis. Subsequent investigation of pyroptosis markers revealed that while total gasdermin D (GSDMD) protein levels remained largely unchanged in drug-treated MND1-silenced cells, its active N-terminal fragment (N-GSDMD) was significantly elevated, accompanied by a pronounced increase in the inflammasome component NLRP3 (Fig. 2I). In stark contrast, MND1-overexpressing cell lines displayed the converse trend, wherein MND1 overexpression conferred significant resistance to PANoptosis induced by gemcitabine or oxaliplatin treatment (Fig. 2H).

Collectively, these findings indicate that MND1 overexpression suppresses PANoptosis in lung adenocarcinoma cells, whereas MND1 knockout synergistically enhances PANoptosis activation in combination with gemcitabine.

### *MND1* promotes stemness of LUAD cell lines in vitro

**Fig. 3:**
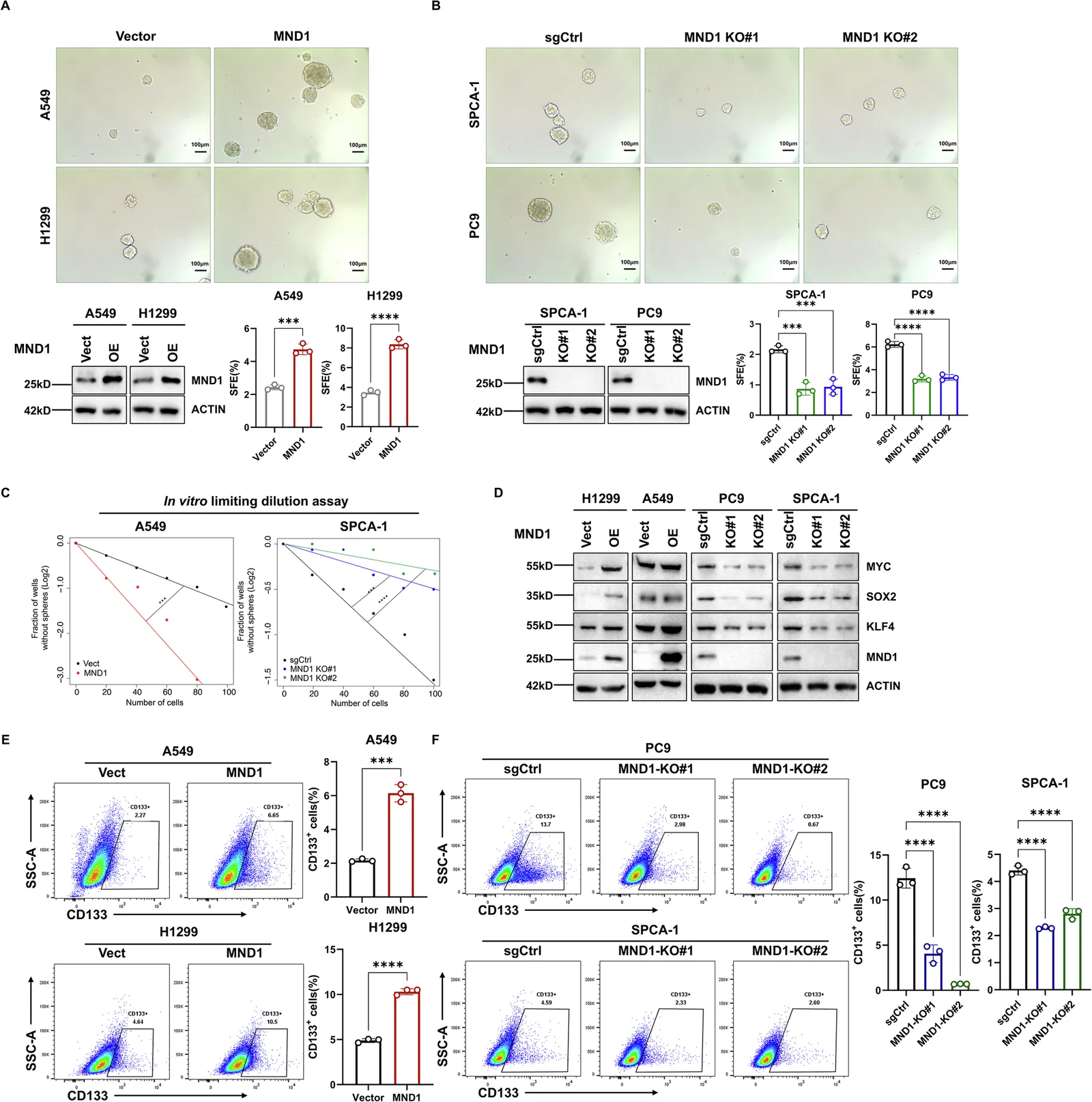
MND1 maintains the stemness phenotype of LUAD cells. **A** Sphere formation experiment in A549 and H1299 cells overexpressing MND1. **B** Sphere formation experiment of PC9 and SPCA-1 cells under conditions of MND1 depletion versus control. **C** In vitro limiting dilution experiments were conducted to assess the self-renewal ability of stably overexpressing or knocking out MND1 cell lines. **D** Western blotting analysis detected MYC, KLF4, and SOX2 in MND1-overexpressing and depletion cells. **E**, **F** Flow cytometry was employed to determine the proportion of CD133+ cells in stable overexpression (**E**) or knockout (**F**) MND1 cell lines. Data in the graphs are presented as mean ± SD. All data in bar graphs were assessed by two-tailed Student’s t-test or one-way ANOVA with multiple comparisons. *P < 0.05, **P < 0.01, ***P < 0.001, ****P < 0.0001.

**Fig. 4:**
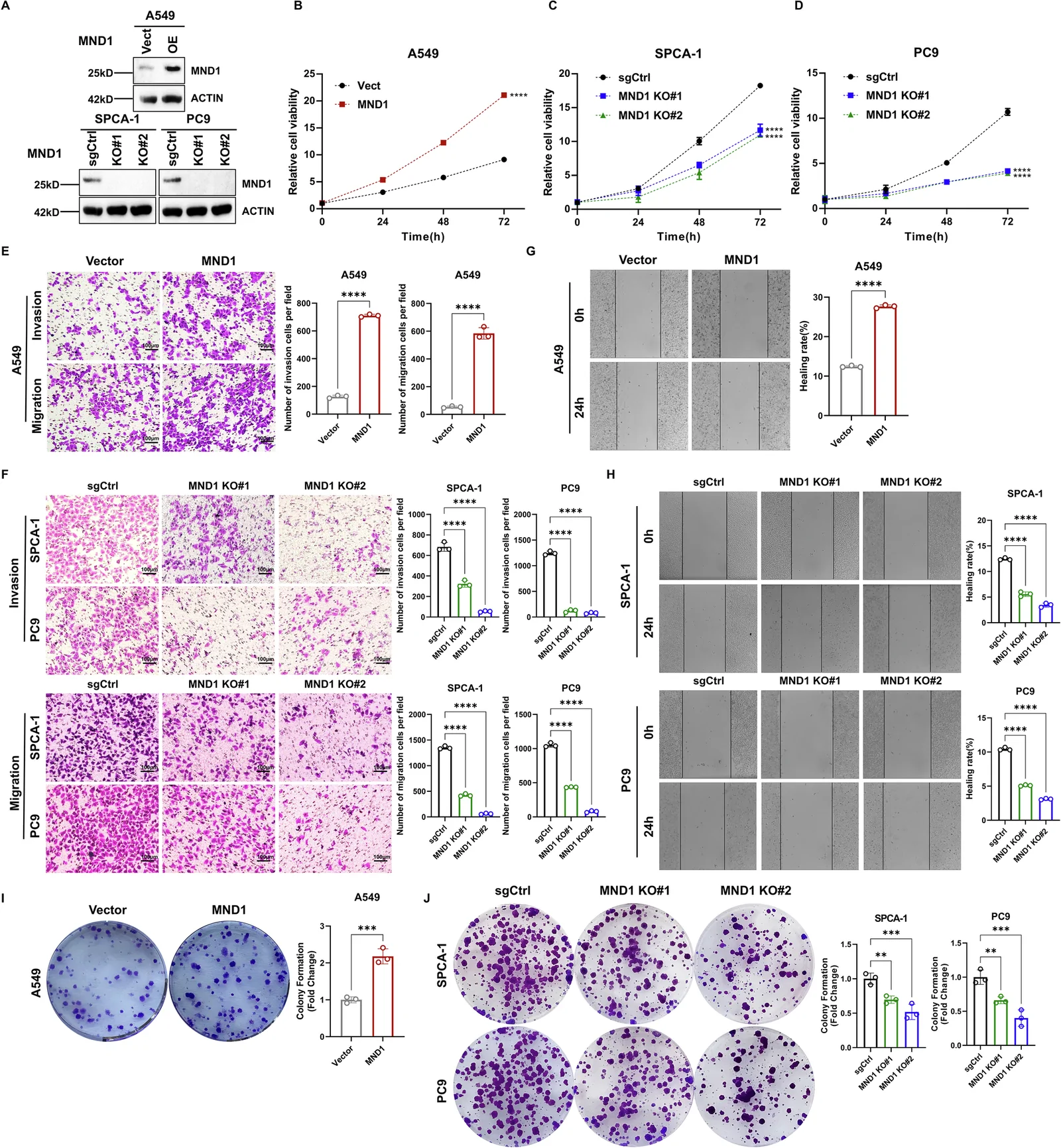
MND1 promotes the proliferation, migration, and invasion of LUAD cells. **A** Western blot analysis was employed to assess the expression levels of the MND1 protein in LUAD cell lines. **B** CCK-8 assay was utilized to assess the proliferation capability of the A549-MND1 cell line. **C** CCK-8 assay was utilized to assess the proliferation capability of the SPCA-1-KO-MND1 cell line. **D** CCK-8 assay was utilized to assess the proliferation capability of the PC9-KO-MND1 cell line. **E** Transwell assay was employed to evaluate the migration and invasion of the A549-MND1 cell line. **F** Transwell assay was employed to evaluate the migration and invasion of the SPCA-1/PC9-KO-MND1 cell line. **G** Wound healing experiment was conducted to evaluate the wound healing capacity of the A549-MND1 cell line. **H** Wound healing experiment was conducted to evaluate the wound healing capacity of the SPCA-1/PC9-KO-MND1 cell line. **I** Colony formation assay was performed to assess the proliferation ability of the A549-MND1 cell line. **J** Colony formation assay was performed to assess the proliferation ability of the SPCA-1/PC9-KO-MND1 cell line. Data in the graphs are presented as mean ± SD. All data in bar graphs were assessed by two-tailed Student’s t-test or one-way ANOVA with multiple comparisons. **P < 0.01, ***P < 0.001, ****P < 0.0001.

To further elucidate the potential impact of *MND1* on the stemness properties of LUAD, we conducted functional investigations utilizing MND1-manipulated cellular models. Tumorsphere formation assays and in vitro limiting dilution analyses [37] revealed that *MND1* overexpression enhanced tumorsphere formation and self-renewal capacity of LUAD cells (Fig. 3A, C). Conversely, *MND1* depletion attenuated these functionalities (Fig. 3B, C). Key stem cell-associated transcription factors, including KLF4, MYC, and SOX2, which are essential for preserving self-renewal capacity and pluripotency of pluripotent stem cells [49]. exhibited elevated upon MND1 overexpression, whereas their expression was notably suppressed upon MND1 deletion (Fig. 3D, Fig. S3A). The utilization of *CD133* and *CD44* markers for identifying CSC functional populations [50] revealed an increase in *CD133*- and *CD44*-positive cells in LUAD cells upon *MND1* overexpression, contrasting with a decrease observed upon *MND1* knockdown (Fig. 3E, F, Fig. S3B). To further delineate the physiological function of MND1 in lung adenocarcinoma, CCK8 assays were conducted, demonstrating that MND1 overexpression markedly promoted the proliferation of lung adenocarcinoma cells (Fig. 4B). Conversely, ablation of MND1 led to a notable decrease in cell proliferation (Fig. 4C, D). Moreover, Transwell migration and invasion assays, along with wound healing assays, demonstrated that MND1 overexpression significantly promoted the migration and invasion capacities of LUAD cells (Fig. 4E, G), while MND1 deficiency impaired these abilities. (Fig. 4F, H). Furthermore, colony formation assays corroborated *MND1’s* promotion of LUAD cell colony-forming potential (Fig. 4I, J). Cumulatively, these findings underscore *MND1’s* propensity to foster the stemness phenotype of LUAD cells, concomitant with its role in promoting cell proliferation, migration, and invasion.

### *MND1* regulates the stemness of LUAD cells through *RCOR2*

To demonstrate the molecular mechanism underlying the influence of MND1 on cancer cell stemness, we conducted RNA sequencing on SPCA-1 cells with stable MND1 knockout compared to controls. Genes whose mRNA levels undergo drastic downregulation upon *MND1* loss may be considered potential targets positively regulated by *MND1*. 458 genes (log2FC < -1, *p* < 0.05) were downregulated in the absence of MND1, of which 16 overlapped with stemness-related genes in the turquoise module (Fig. S4A). Among these, NEIL3, RCOR2, RHEBL1, SAP30, and TEX19 showed the strongest correlation with mRNAsi in 500 LUAD TCGA samples (Fig. S4B). Validation via RT-qPCR and Western blot analysis confirmed that RCOR2 expression was positively modulated by MND1 at both the mRNA and protein levels (Fig. S4C, D), indicating that RCOR2 is a potential downstream target of MND1. *MND1* may maintain the stemness of LUAD cells by regulating *RCOR2*.

**Fig. 5:**
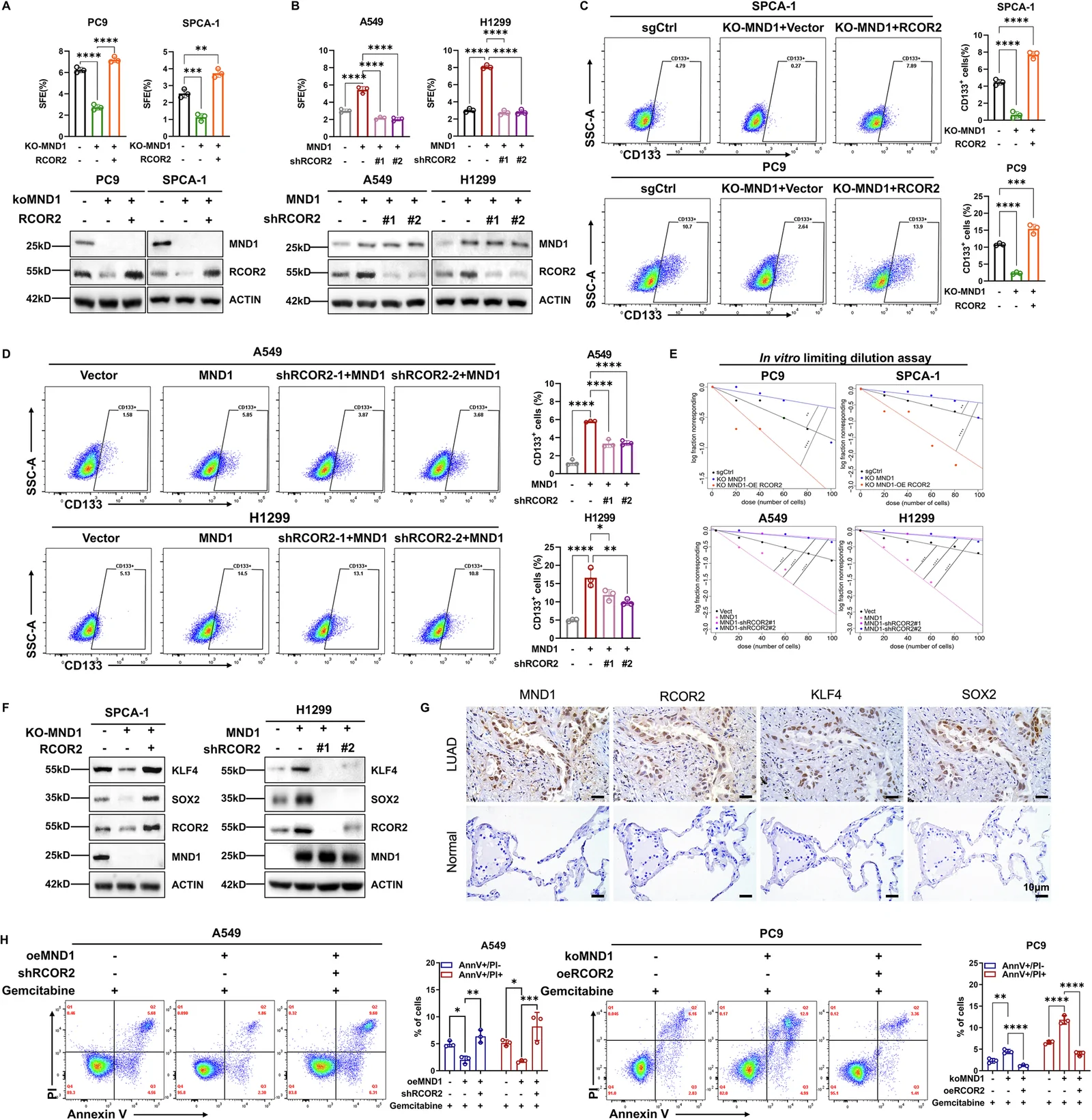
MND1 regulates the stemness of LUAD cells through RCOR2. **A** Tumor sphere formation experiment detects the size of tumor spheres formed by MND1 knockout and RCOR2 overexpressing cells. **B** Tumor sphere formation experiment detects the size of tumor spheres formed by MND1 overexpressing and RCOR2 knockdown cells. **C** Flow cytometry was employed to detect the proportion of CD133+ cells in MND1-knockout and RCOR2-overexpressing cells. **D** Flow cytometry was used to detect the proportion of CD133+ cells in MND1-overexpressing and RCOR2-knockdown cells. **E** Limiting dilution in vitro was used to detect the self-renewal ability. **F** Western blot was used to determine the expression of KLF4 and SOX2 in MND1 knockout and RCOR2 overexpression cells, as well as in MND1 overexpression and RCOR2 knockdown cells. **G** Immunohistochemical staining for the indicated proteins derived from human LUAD and its adjacent normal tissue. **H** Flow cytometry assays were used to examine the apoptosis of cells treated with gemcitabine (10 μg/mL) for 24 h. Data in the graphs are presented as mean ± SD. All data in bar graphs were assessed by two-tailed Student’s t-test or one-way ANOVA with multiple comparisons. *P < 0.05, **P < 0.01, ***P < 0.001, ****P < 0.0001.

*RCOR2* has been reported to operate as a stemness transcription factor [51], but whether *MND1* regulates stemness in LUAD cells via *RCOR2* is unknown. Therefore, we performed rescue experiments and suppression experiments. The results from tumor sphere formation, flow cytometry, and in vitro limiting dilution assays demonstrate that attenuated *RCOR2* expression effectively impeded tumor sphere formation capacity, the proportion of CD133^+^ cells, and the self-renewal proficiency of LUAD cells induced by *MND1*. While reintroduction of RCOR2 significantly alleviated the reduction in tumor sphere formation, decrease in CD133^+^ cell proportion, and impairment of self-renewal capacity induced by MND1 depletion in LUAD cells. (Fig. 5A–E, Fig.S5A, B). Western blot experiment unveiled that the depletion of RCOR2 abrogated the elevated expression levels of KLF4 and SOX2 instigated by MND1 overexpression. Conversely, the overexpression of RCOR2 restored the diminished expression levels of KLF4 and SOX2 resulting from MND1 deficiency (Fig. 5F). All of these outcomes indicate that *MND1* promotes the stemness properties of LUAD cells by regulating *RCOR2*. To further confirm the correlation between MND1, RCOR2, and the stemness properties of LUAD cells, we conducted immunohistochemical (IHC) staining on 30 paired LUAD tissues. The results showed that MND1, RCOR2, KLF4, and SOX2 were predominantly localized in the cell nucleus, and both MND1 and RCOR2 were positively correlated with KLF4 and SOX2, supporting the role of MND1 in promoting stemness through RCOR2 in LUAD cells (Fig. 5G, Fig. S5C). Furthermore, to validate the functional interplay among MND1, RCOR2, and PANoptosis in LUAD cells, we conducted rescue experiments. Western blot and flow cytometry analyses revealed that RCOR2 knockdown effectively abrogated MND1-mediated resistance to gemcitabine-induced PANoptosis. Conversely, ectopic expression of RCOR2 significantly attenuated PANoptosis triggered by MND1 deficiency in LUAD cells (Fig. 5H, Fig. S5D). These findings collectively demonstrate that MND1 modulates stemness and PANoptosis in LUAD cells, at least in part, through the RCOR2-dependent pathway.

### MND1 modulates the stability of RCOR2 mRNA via PABPC1

**Fig. 6:**
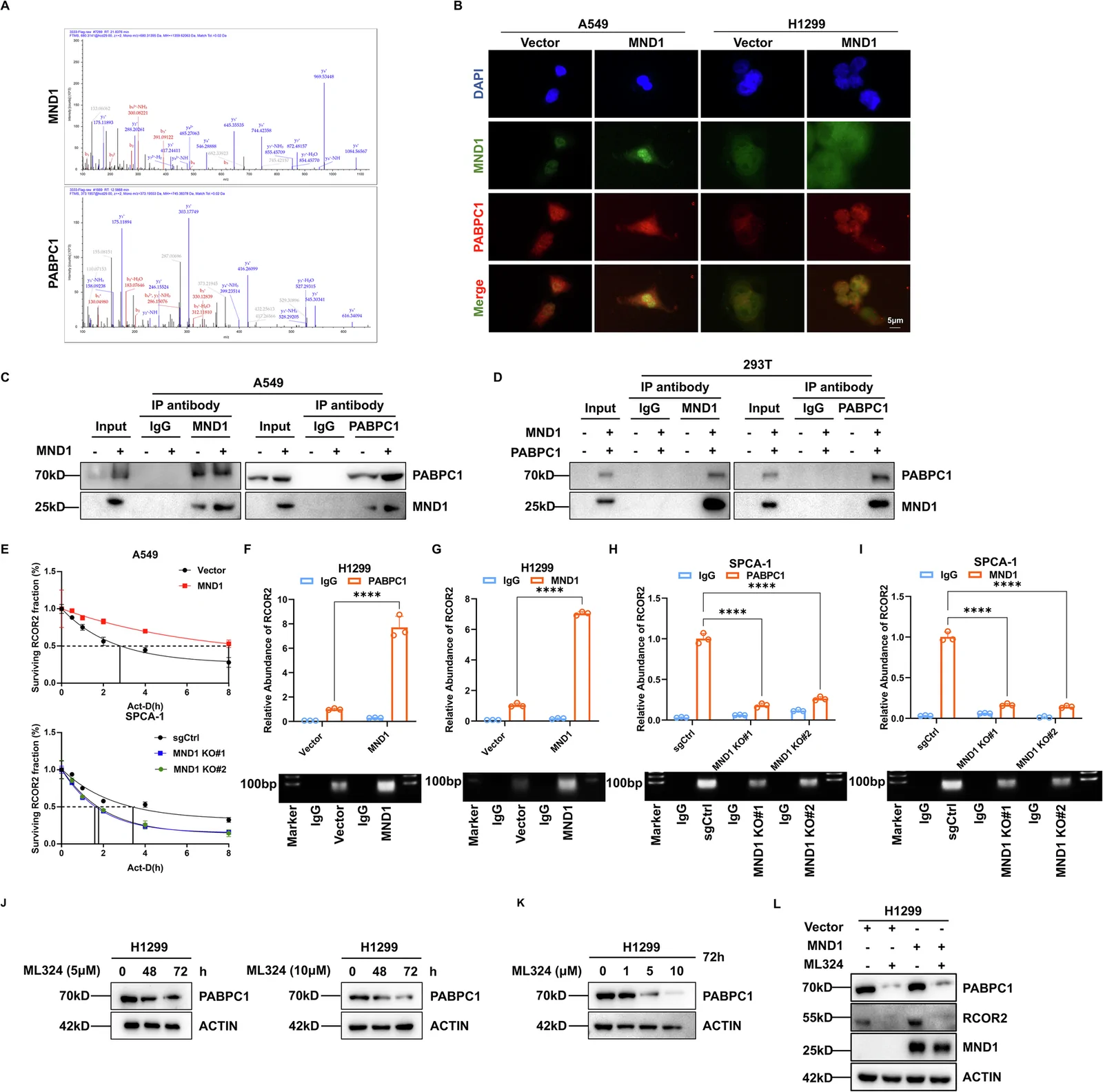
MND1 modulates the stability of RCOR2 mRNA via PABPC1. **A** Mass spectrometry analysis shows that MND1 interacts with PABPC1. **B** Immunofluorescence and laser confocal reveal colocalization of MND1 and PABPC1. **C**, **D** Co-IP shows endogenous (**C**) and exogenous (**D**) interactions of MND1 with PABPC1. **E** Actinomycin D (10 μg/mL) was applied to LUAD cells transfected with MND1 over-expressing or depletion. RNA was extracted at indicated time points to detect the mRNA level of RCOR2. **F**, **G** RIP assay in H1299 cells showed that MND1 and PABPC1 bound directly to RCOR2 mRNA; anti-IgG was employed as a negative control. **H**, **I** RIP assay in SPCA-1 cells shows that MND1 deletion reduces MND1 and PABPC1 binding to RCOR2 mRNA; anti-IgG was employed as a negative control. **J** Western blot analysis was employed to assess the expression of PABPC1 in H1299 cells following treatment with varying doses of ML324 for 72 h. **K** Western blot analysis was conducted to evaluate the expression of PABPC1 in H1299 cells at different time points following treatment with ML324. **L** Western blot experiment was performed to evaluate the protein expression levels of PABPC1 and RCOR2 in cells treated with 10 μM ML324 for 72 hours. Data in the graphs are presented as mean ± SD. All data in bar graphs were assessed by two-tailed Student’s t-test or one-way ANOVA with multiple comparisons. ****P < 0.0001.

To investigate how MND1 influences RCOR2, we performed co-immunoprecipitation (Co-IP) assays followed by mass spectrometry in A549 cells overexpressing MND1. GSEA analysis identified that proteins interacting with MND1 were predominantly enriched in transcription-associated pathways, notably including mRNA 3’-UTR binding and regulation of RNA metabolism (Fig. S6A). Focusing on the top 50 proteins exhibiting the strongest interactions with MND1, further protein-protein interaction analysis identified PABPC1, along with ACT8, HNRNPK, NCL, and NPM1, as key interacting partners (Fig. S6B). Of particular interest, PABPC1 emerged as the most interconnected RNA-binding protein, known to engage with the Poly(A) signal located at the 3’-UTR site and interact with translation regulators and mRNA modification factors, thereby contributing to the maintenance of mRNA stability. Based on this, we postulated that MND1 modulates *RCOR2* through its interaction with PABPC1. Mass spectrometry peak analyses corroborated the physical association between MND1 and PABPC1 (Fig. 6A). Immunofluorescence staining further confirmed the colocalization of MND1 and PABPC1, with heightened colocalization observed upon MND1 overexpression (Fig. 6B). Immunoprecipitation confirmed the interaction of endogenous MND1 and PABPC1 in A549 cells (Fig. 6C), as well as in exogenous MND1 and PABPC1 in 293 T cells (Fig. 6D). Based on the above findings, we propose the hypothesized that MND1 may stabilize RCOR2 mRNA via its interaction with PABPC1.

Treatment of *MND1*-overexpressing cells with the transcriptional inhibitor actinomycin D revealed a prolonged half-life of *RCOR2* mRNA, while depletion of *MND1* resulted in diminished stability of *RCOR2* mRNA (Fig. 6E). Subsequent RIP experiments demonstrated significant enrichment of *PABPC1* and *MND1* on *RCOR2* mRNA upon *MND1* overexpression (Fig. 6F, G), whereas depletion of *MND1* inhibited the enrichment of *PABPC1* and *MND1* on *RCOR2* mRNA (Fig. 6H, I). Furthermore, we treated H1299 cells with the PABPC1 inhibitor ML324 [52] at different concentrations for 0 h, 48 h, and 72 h. The results demonstrate that ML324 inhibits PABPC1 expression in a time- and dose-dependent manner (Fig. 6J). Furthermore, ML324 not only suppresses the expression of PABPC1 but also inhibits the expression of RCOR2 simultaneously (Fig. 6K). Subsequent mRNA stability experiments and RIP assays, conducted upon joint interference with *MND1* and *PABPC1*, revealed that silencing of PABPC1 attenuated the supportive role of *MND1*, whereas *PABPC1* overexpression rescued the inhibitory phenotype induced by *MND1* deletion (Fig. S6C). RIP experiments demonstrated that PABPC1 inhibitor ML324 inhibited the enrichment of *PABPC1* and *MND1* on *RCOR2* mRNA, while complementation of *PABPC1* increased *MND1* and *PABPC1* binding to *RCOR2* mRNA (Fig. S6D–G). Subsequently, using the RBPDB database, we identified a potential binding site for PABPC1 in the 3’-UTR of *RCOR2* mRNA at the 5’-AAAAA-3’ sequence (Fig. S7D). Dual-luciferase assays were further conducted employing reporter genes carrying either the wild-type *RCOR2* 3’-UTR or a truncated form of the *RCOR2* 3’-UTR with deleted PABPC1 binding sites (nucleotides 611-615/618-622) (Fig. S6I). The overexpression of MND1 and PABPC1 significantly increased luciferase activity in HEK293 cells transfected with the wild-type *RCOR2* 3’-UTR, while the truncated 3’-UTR did not show significant effects (Fig. S6J). These findings suggest that MND1 regulates RCOR2 mRNA stability through PABPC1.

### *MND1* inhibition reduces tumorigenicity in LUAD

**Fig. 7:**
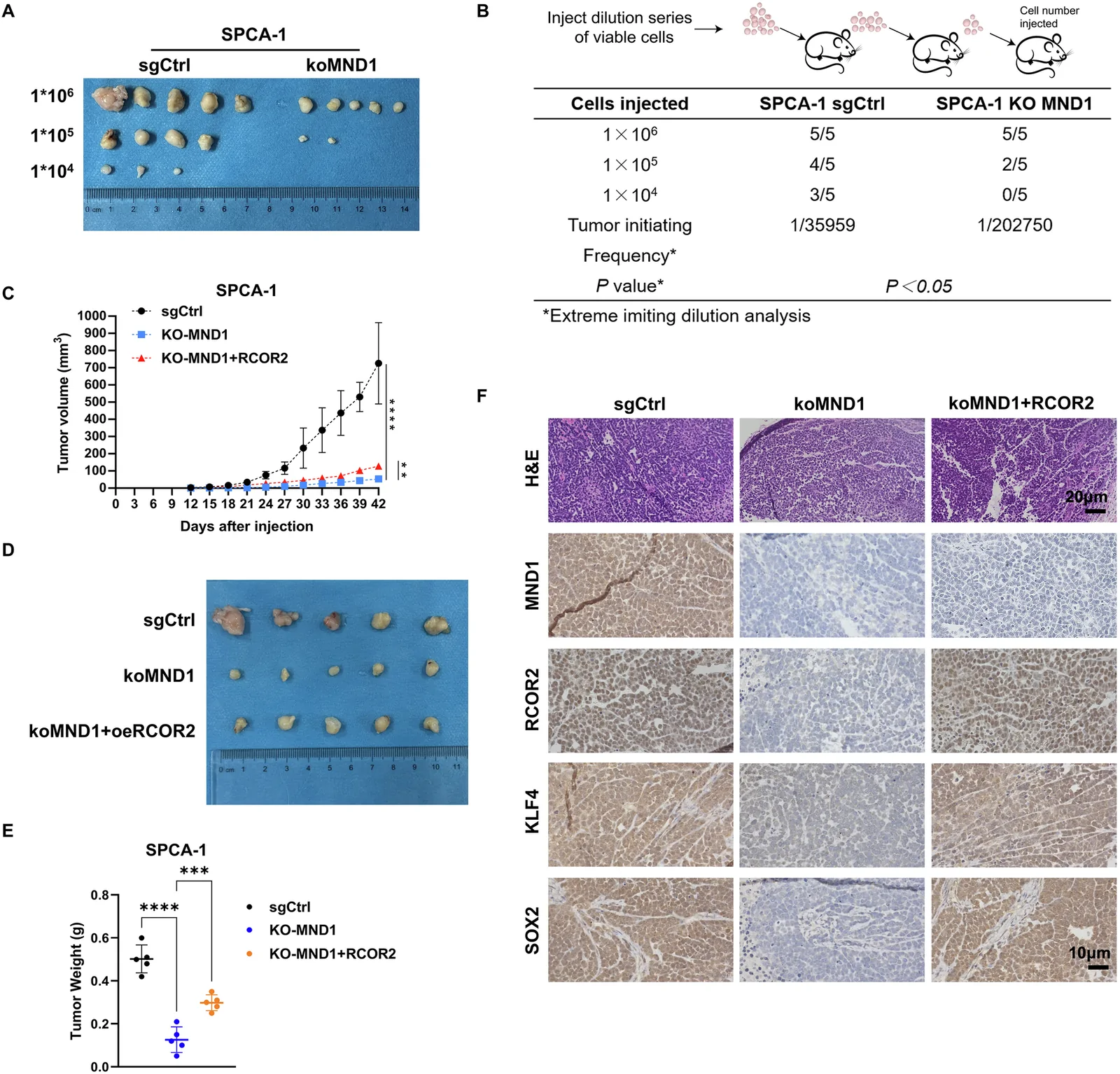
Inhibition of MND1 suppresses the tumorigenic potential of LUAD cells *in vivo.* **A**, **B** In vivo limiting dilution experiment of SPCA-1 cells with or without MND1-depletion. SPCA-1 cells were injected subcutaneously into NOD-SCID mice at three different doses (1 × 10⁴, 1 × 10⁵, and 1 × 10⁶ cells per injection). Tumor formation was monitored, and the tumor-initiating frequency was calculated using extreme limiting dilution analysis (ELDA) software. **C**–**E** A xenograft tumor model was established in nude mice, with tumor volumes quantitatively assessed at predetermined time intervals. **F** Hematoxylin and eosin, as well as immunohistochemical staining for the target protein, was performed on a xenograft derived from MND1-depleted, MND1-depleted and RCOR2-overexpression SPCA-1 cells or control cells. Data in the graphs are presented as mean ± SD. All data in bar graphs were assessed by two-tailed Student’s t-test or one-way ANOVA with multiple comparisons. **P < 0.01, ***P < 0.001, ****P < 0.0001.

To assess the contribution of MND1 in tumorigenicity, we conducted an in vivo limiting dilution assay (LDA) by injecting athymic nude mice with varying numbers of SPCA-1 cells, either with MND1 knockout or control. Over a 42-day observation period, control and *MND1*-knockout SPCA-1 cells exhibited a gradual decrease in tumorigenicity with a reduction in the number of injected cells. In the low cell quantity group (1 × 10^4^), the control group cells retained tumorigenicity, while the knockout group cells did not exhibit tumorigenic potential (Fig. 7A). Knockdown of *MND1* resulted in a reduced frequency of tumor formation in vivo at low dilution levels (Fig. 7B). To ascertain whether the *MND1*-*RCOR2* axis also operates in the in vivo progression of lung adenocarcinoma, we established a xenograft nude mouse model and evaluated the formation of xenograft tumors in this animal model. We observed that tumors formed in nude mice inoculated with SPCA-1-ko*MND1* cells exhibited markedly smaller tumor volume, dimensions, and weight compared to mice injected with control cells. However, the restoration of *RCOR2* rescued the *MND1* deficiency-mediated inhibition of tumor growth (Fig. 7C–E). Moreover, in comparison to control cell biopsies from xenograft tumors, MND1 deficiency in SPCA-1 biopsies impaired the protein staining of KLF4 and SOX2, while the overexpression of RCOR2 restored the expression of KLF4 and SOX2 (Fig. 7F). The summarized outcomes of in vivo functional analyses conducted through animal experiments align with our in vitro functional findings and substantiate the involvement of the *MND1*-*RCOR2* axis in the progression of in vivo LUAD.

## Discussion

This investigation reveals that *MND1* facilitates the proliferation and invasion of LUAD by suppressing PANoptosis and augmenting cellular stemness. GSEA and GSVA analyses suggest a potential association between *MND1* and both tumor stemness and PANoptosis. Indeed, the overexpression of *MND1* was observed to enhance stemness traits in cancer cells. Notably, compared with the control group, *MND1* overexpression significantly decreased the occurrence of apoptosis, necrosis, and pyroptosis, collectively termed PANoptosis. In contrast, *MND1* depletion yielded opposite outcomes. Interestingly, upon treatment with gemcitabine, cancer cell lines deficient in *MND1* exhibit significantly heightened PANoptosis levels relative to control cells. During this process, the expression levels of apoptosis-associated proteins, necrosis-associated proteins, and pyroptosis-associated proteins are significantly upregulated. These results indicate that *MND1* promotes tumor chemoresistance to gemcitabine through the suppression of PANoptosis in cancer cells. As far as we know, this study is the earliest evidence elucidating *MND1*’s role in modulating PANoptosis to augment gemcitabine resistance, with RCOR2 serving as the critical downstream mediator that bridges MND1 function to these two core tumor hallmarks.

Here, we identified that MND1 significantly promotes cancer cell stemness while concurrently suppressing PANoptosis. *MND1* is widely recognized in meiotic homology search and recombination [53]. In various cancers, MND1 has been established to function as a promoter of tumorigenesis. The preceding investigations have substantiated the involvement of *MND1* in promoting the tumorigenic growth of pulmonary adenocarcinoma [20]. Furthermore, a close association has been established between *MND1* and immune infiltration as well as cell cycle regulation in renal clear cell carcinoma [22]. Overexpression of *MND1* has been related to enhanced proliferation and metastasis in liver cancer [21]. Nevertheless, the specific impact of *MND1* on the regulation of cancer stemness remains elusive. In this study, we reveal that *MND1* overexpression upregulates pluripotency transcription factors, including *KLF4* and *SOX2*, as well as stem cell markers such as *CD133* expression. Notably, we identify *MND1* as a regulator of the pivotal stemness driver, *RCOR2*—an emerging oncogenic molecule that exerts its function primarily through transcriptional regulation, with well-documented implications in tumor progression across multiple cancer types [54–56].

As a core component of the REST co-repressor complex, RCOR2 exerts its transcriptional regulatory function by recruiting histone deacetylases (HDACs) and other epigenetic modifiers to target gene promoters, thereby modulating the transcription of genes involved in stemness maintenance, cell cycle progression, and cell death pathways [25, 55, 57]. Beyond its canonical role in transcriptional repression, recent drug discovery efforts have identified small molecules that directly target CoREST complexes, highlighting the translational potential of modulating this pathway. For example, Corin, a bifunctional inhibitor of LSD1 and HDAC1/2, disrupts CoREST enzymatic functions and has been shown to reprogram chromatin states and inhibit tumor progression in preclinical models [58–60]. Similarly, the small molecule UM171 acts as a molecular glue that facilitates CUL3–KBTBD4 E3 ligase–mediated ubiquitination and degradation of HDAC1/2 and other CoREST core components, demonstrating a novel strategy for selective CoREST complex disruption with therapeutic implications [61, 62].

In endometrial cancer, RCOR2 overexpression has been associated with advanced tumor stage, lymph node metastasis, and poor patient prognosis [55]. In addition, RCOR2 suppresses CIITA expression via HDAC1/2-mediated deacetylation of histone H4 at lysine 16 (H4K16), resulting in transcriptional silencing of MHC class II molecules in tumor cells. This downregulation compromises antigen presentation capacity, impairs CD4⁺ and CD8⁺ T cell-mediated immune surveillance, and ultimately facilitates tumor immune evasion [54]. Notably, RCOR2 also induced Wnt/β-catenin activation and tumor stemness by inhibiting RNF43 expression, further supporting its oncogenic role in our target cancer type [54].

Although CoREST family complexes have traditionally been considered predominantly transcriptional repressors, accumulating evidence reveals a functional heterogeneity among their components. Recent studies have demonstrated that RCOR2 can also play non‑canonical transcriptional activation roles in certain cancer contexts: for example, in MYCN‑amplified adrenergic neuroblastoma, RCOR2 is driven by super‑enhancers and participates in core regulatory circuitry by facilitating enhancer‑promoter interactions that sustain expression of oncogenic gene networks, a behavior distinct from its canonical repressive function [63, 64].

In breast cancer, RCOR2 cooperates with the transcription factor SNAIL to promote epithelial-mesenchymal transition (EMT) and distant metastasis [65]. Additionally, RCOR2 has been shown to regulate cell cycle progression and inhibit apoptosis in glioblastoma, further supporting its oncogenic function [26]. These findings collectively underscore the critical importance of RCOR2 in tumor development, aligning with our observation that RCOR2 acts as a key downstream effector of MND1 to promote LUAD stemness. Moreover, our findings demonstrate that MND1 interacts with the RNA-binding protein PABPC1 to maintain the stability of *RCOR2* mRNA, forming a novel regulatory axis that links mRNA post-transcriptional regulation to cancer stemness and PANoptosis-a regulatory pattern that complements RCOR2’s intrinsic transcriptional function and expands our understanding of its oncogenic mechanism in LUAD.

Subsequently, we investigated the mechanism by which *MND1* promotes the stemness of LUAD cells and its functional connection to PANoptosis. Through RNA sequencing, we identified *RCOR2* as a downstream target gene of *MND1*. Previous studies have reported that *RCOR2* is crucial for maintaining stem cell status throughout the cell cycle and is essential for pluripotency during reprogramming into fibroblastic lineages [25, 66]. In the transcriptional context, RCOR2 modulates the expression of pluripotency genes by balancing the recruitment of HDACs, thereby maintaining the stem cell transcriptional program [67]. Therefore, we speculate that *RCOR2* is a stemness-related transcription factor that can directly affect cancer stemness. Interestingly, we found that *MND1* can positively regulate the expression of *RCOR2*. In our study, overexpression of *RCOR2* in *MND1*-knockout cell lines rescued the stemness inhibition caused by *MND1* deficiency. Conversely, knocking down *RCOR2* in MND1-overexpressing cell lines suppressed the enhancement of stemness induced by *MND1*. This is consistent with previous findings directly confirming that RCOR2 is a critical mediator linking MND1-dependent stemness regulation. This mechanistic connection is further supported by the fact that RCOR2 binds to the promoters of key stemness related genes such as *Nanog*, *Oct4*, and *Sox2* [51], where it exerts transcriptional control to sustain the stemness phenotype, and these pluripotency factors have been reported to cross-talk with PANoptosis-related signaling pathways. For example, SOX2 can repress the activation of the NLRP3 inflammasome and downregulate the expression of cleaved caspase-3 and phosphorylated MLKL [68–70]. Thus, we hypothesize that the MND1-PABPC1-RCOR2 axis coordinately regulates stemness and PANoptosis by modulating the expression of these downstream molecules. Specifically, MND1-PABPC1-mediated stabilization of RCOR2 mRNA enhances the expression of Nanog/Oct4/SOX2, which in turn suppresses the activation of PANoptosis-related pathways, ultimately conferring stemness and chemoresistance to LUAD cells. In this process, the transcriptional activity of RCOR2 serves as the central link between MND1-mediated post-transcriptional regulation and the functional outcomes of stemness and PANoptosis.

According to earlier research, *RCOR2*, and poly(A)-RNA co-localize at nuclear speckles, where poly(A)-RNA forms fibrillar structures and decorates the edges-a subnuclear region closely associated with pre-mRNA processing and transcriptional regulation, further supporting the functional relevance of RCOR2 in mRNA stability and transcriptional control [71]. At the same time, we found that PABPC1 interacts with MND1. Therefore, we hypothesized that MND1 interacts with PABPC1 to maintain the stability of *RCOR2* mRNA. PABPC1 is a classic poly(A) tail-binding protein. Translation start and mRNA decay regulation are two of PABPC1’s main roles [28]. PABPC1 accompanies mRNA throughout its entire lifecycle-from initial nuclear synthesis to eventual degradation [28]. It has been demonstrated by numerous earlier research that PABPC1 is crucial for the development of tumors [72–74]. For instance, PABPC1 synergizes with IGF2BP1 in endometrial cancer to enhance the mRNA stability of PEG10, thereby promoting tumorigenesis [75]. PABPC1 has been shown to engage in functional interactions with PLCB1 and PI3K in cholangiocarcinoma, thereby potentiating PLCB1-driven EMT and chemoresistance through the PI3K/AKT/GSK3/Snail signaling axis [76]. Our RIP studies revealed that PABPC1 is an *RCOR2* mRNA-binding protein that maintains the stability of *RCOR2* mRNA. Furthermore, the PABPC1 inhibitor ML324 can prevent PABPC1 from binding to *RCOR2* mRNA, confirming that the MND1-PABPC1 interaction is essential for both RCOR2 stabilization and PANoptosis regulation. Furthermore, we observed that the complex formed by MND1 and PABPC1 primarily exerts its function through binding to the 3’-UTR of *RCOR*2, a region critical for mRNA stability regulation. Our research results suggest that the MND1-PABPC1-RCOR2 axis is a key regulatory module, contributes to the maintenance of RCOR2 mRNA stability, which in turn promotes the stemness properties of LUAD.

In conclusion, we performed a mechanistic investigation demonstrating that *MND1* preserves the mRNA stability of *RCOR2* through its interaction with PABPC1, forming a novel MND1-PABPC1-RCOR2 regulatory axis that coordinately fosters stemness in LUAD cells and suppressing PANoptosis. This axis bridges two fundamental hallmarks of tumor progression-dysregulated stem cell function and impaired cell death, provides a molecular explanation for MND1-mediated gemcitabine resistance. Our study underscores the pivotal role of *MND1* in cancer stemness and PANoptosis, highlighting their potential as prognostic biomarkers and therapeutic targets for LUAD. Ultimately, our results indicated that targeting the MND1-PABPC1-RCOR2 axis may simultaneously inhibit tumor stemness and restore PANoptosis sensitivity, thereby enhancing chemotherapeutic efficacy and preventing tumor recurrence.

Notably, our in vivo tumorigenesis assays revealed a partial phenotypic rescue when RCOR2 was re-expressed in MND1 knockout LUAD cells, while RCOR2 overexpression significantly increased tumor volume and weight compared to the MND1 KO group, the rescued tumors remained substantially smaller than those in the wild-type control group. This partial rescue phenotype, rather than negating the functional relevance of the MND1-PABPC1-RCOR2 axis. First, the in vivo tumor microenvironment is far more complex than the in vitro cell-autonomous model. Tumor growth in nude mice is coordinately regulated by intrinsic tumor cell properties and extrinsic factors, including tumor-associated stromal cells, residual immune cells, and paracrine signaling networks. For instance, MND1 depletion has been reported to alter immune infiltration patterns in solid tumors [22], and the residual anti-tumor immune microenvironment in MND1 KO mice may have limited the tumor-promoting effect of RCOR2 rescue, contributing to the incomplete restoration of tumor size. Second, MND1 exerts its oncogenic function through multiple parallel downstream pathways, with the MND1-PABPC1-RCOR2 axis being a key validated mediator rather than the sole regulatory module.

Beyond regulating RCOR2-dependent stemness and PANoptosis, MND1 is known to modulate cell cycle progression, metabolic reprogramming, and immune evasion [22, 77]. Thus, single restoration of RCOR2 can only rescue the oncogenic functions mediated by this specific axis, rather than compensating for all MND1 deficiency-induced functional losses. Critically, the statistically significant increase in tumor growth in the RCOR2-rescued group relative to the MND1 KO group directly validates that the MND1-PABPC1-RCOR2 axis is a bona fide, functionally important mediator of MND1’s oncogenic activity in LUAD. Collectively, these results underscore the multifaceted regulatory network of MND1 in tumorigenesis and reinforce the clinical relevance of targeting the MND1-PABPC1-RCOR2 axis for LUAD therapy, even as they reflect the inherent complexity of in vivo biological systems.

## Conclusion

**Fig. 8:**
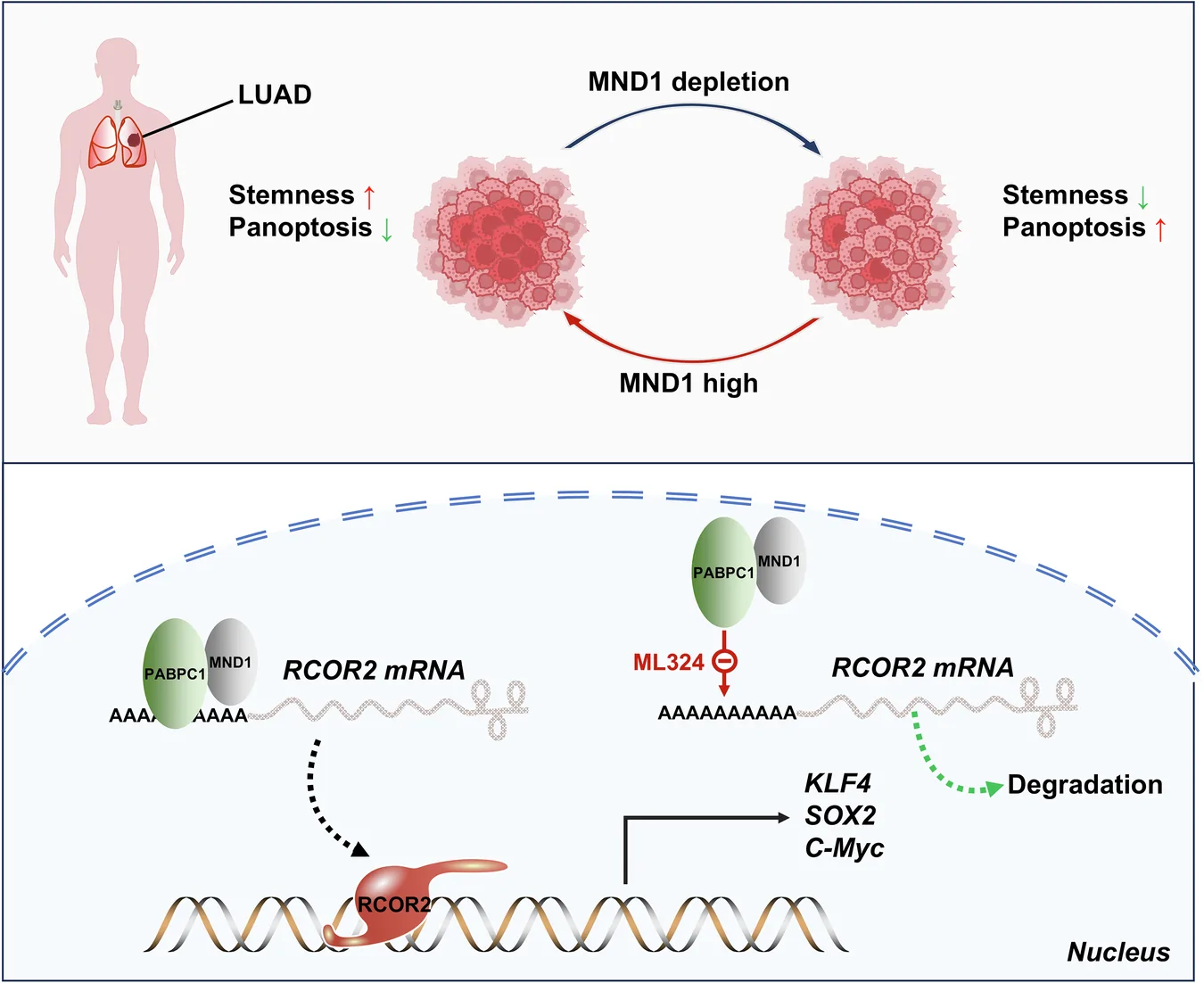
Schematic illustration depicting the regulatory role of MND1 in lung adenocarcinoma (LUAD). This model summarizes the key findings of the study, highlighting that MND1 modulates two critical biological processes in LUAD: (1) the maintenance of stemness properties in cancer cells, which contributes to tumor initiation, progression, and therapeutic resistance; and (2) the regulation of PANoptosis, a pro-inflammatory programmed cell death pathway involved in antitumor immunity and disease pathogenesis. Mechanistically, MND1 interacted with the 3’-UTR of RCOR2 mRNA by recruiting PABPC1 to enhance the stability of RCOR2 mRNA. As a crucial component of the pluripotency regulatory network, the stable expression of RCOR2 is essential for maintaining tumor cell stemness. Our findings suggest MND1 acts as an oncogene in LUAD by maintaining RCOR2 mRNA stability, promoting stemness and inhibiting PANoptosis.

In summary, we have validated the MND1-PABPC1-RCOR2 axis as a regulatory pathway for cancer stemness and PANoptosis, which may function as a molecular biomarker for LUAD and a potential therapeutic target for therapeutic intervention in cancer treatment (Fig. 8).

## Supplementary information

Supplementary material

## Data Availability

The datasets utilized in the present study are accessible through the TCGA (https://xenabrowser.net/datapages/) and GEO databases (https://www.ncbi.nlm.nih.gov/geo/query/acc.cgi?acc=GSE119911).
