# Supplementary material for: MND1 regulates PANoptosis and stemness of lung adenocarcinoma cells by stabilizing RCOR2 mRNA
Source: Cell Death Dis. 2026 Jul 10;17(1):857. doi: 10.1038/s41419-026-09012-5 (PMC13635180; doi:10.1038/s41419-026-09012-5)
Supplement: Supplementary file 1 [file 41419_2026_9012_MOESM1_ESM.docx]

**Supplementary information**

This file contains supplementary methods, supplementary figures S1-S7 and supplementary table S1.

**MND1 Regulates PANoptosis and Stemness of Lung Adenocarcinoma Cells by Stabilizing RCOR2 mRNA**

Lianlian Ouyang^1,2#^, Xingjie Tang^1 #^, Shiyao Jiang^1#^, Yating Liu^3^, Na Liu^4^, Chao Mao^5^, Yuanbing Chen^6^, Ye Fan^7^, Ziyun Zhang^1^, Jingjing Huang^1^, Yongguang Tao^8^, Xing Yu^9,10^, Li Cong^1*^, Yiqun Jiang^1,9*^

1. School of Basic Medical Sciences, Hunan Normal University Health Science Center, Hunan Normal University, Changsha, 410013 Hunan, China.

2. Department of Pathology, Xiangya Hospital, Central South University, Changsha, 410008 Hunan, China

3. Department of Pharmacy, The Third Xiangya Hospital, Central South University, Changsha, 410013, Hunan, China

4. School of Basic Medicine, Central South University, Changsha, Hunan, 410008, China

5. Department of Pathology, Nanjing Drum Tower Hospital, State Key Laboratory of Pharmaceutical Biotechnology, School of Life Sciences, Nanjing University, Nanjing 210023, China

6. Department of Neurosurgery, The Third Xiangya Hospital, Central South University, Changsha, 410013, Hunan, China

7. The Second People's Hospital of Hunan Province/Brain Hospital of Hunan Province, Changsha, 410007 Hunan, China

8. Hunan Key Laboratory of Cancer Metabolism, Hunan Cancer Hospital and The Affiliated Cancer Hospital of Xiangya School of Medicine, Central South University, Changsha, 410078 Hunan, China.

9. Key Laboratory of Model Animals and Stem Cell Biology in Hunan Province, Hunan Normal University School of Medicine; Engineering Research Center of Reproduction and Translational Medicine of Hunan Province; Manufacture-Based Learning and Research Demonstration Center for Human Reproductive Health New Technology of Hunan Normal University, Changsha 410013 Hunan, China.

10. School of Basic Medical Sciences, Hunan Normal University, Changsha, China; Biliary Tract Surgery Laboratory, Department of Hepatobiliary Surgery, Hunan Provincial People's Hospital, the First Affiliated Hospital of Hunan Normal University, Changsha, 410005 Hunan, China.

\# Contribute equally

* Corresponding Author: Li Cong, congli@hunnu.edu.cn; Yiqun Jiang(Lead Contact), jiangyiqun@hunn.edu.cn

**Supplementary Methods：**

1. RNA extraction and qRT‐PCR analysis

Total RNA was extracted by Trizol lysis method. The first-strand cDNA was synthesized using (R223-01, Vazyme, China) reverse transcription kit. Quantitative PCR detection was performed using qPCR mixture on a real-time PCR instrument. The Sangon Biotech (China) synthesized the primers required for this study. Table S1 lists the primer sequences.

2. Western blotting, and immunofluorescence

Cells were lysed for 30min in protein and immunoprecipitation (IP) cell lysis buffer (P0013, Beyotime, China) containing a mixture of 1× protease inhibitor (#C0001, Tsbiochem). Equal quantities of protein lysates were separated by SDS-PAGE electrophoresis and subsequently transferred onto PVDF membranes (Biosharp, Hefei, China). Then incubate the membranes with the primary antibodies overnight at 4 °C, followed by incubation with secondary antibodies (#S0001, Affinity). Protein bands were visualized using ChemistarTM High-sig ECL kit (Vazyme, China). Using chemiluminescence system for image visualization analysis (Tanon-5200, China).

In the cell immunofluorescence assay, 3% paraformaldehyde was first used for fixation, and then 0.5% Triton X-100 was used for permeabilization treatment. (#9002-93-1, Coolaber). Subsequently, cells were incubated in PBS containing 5% BSA for blocking, followed by sequential incubated with primary antibodiesand and fluorescent labeled secondary antibodies (#A23420, #A23210, Abbkine). DAPI (Vector.H-1200) was used for nuclear counterstaining. YO-PRO-1/PI cell apoptosis and necrosis detection kit was used for PANoptosis detection assay. After staining, cells were observed under a fluorescence microscope, followed by corresponding data analysis. The following primary antibodies are used: MND1 (#bs-7799R, Bioss, America), PABPC1 (#10970-1-AP, Proteintech, China).

3. Co‐immunoprecipitation (Co‐IP) and LC-MS/MS

Cells were lysed in IP lysis buffer, and then the cell lysate was incubated overnight with anti-Flag magnetic beads (#KTSM1308, Alpalife, China) at 4°C. Wash the sediment three times, add 5× loading buffer and boil for denaturation. Finally, the samples were analyzed using Western blot method. Oeasy Biotechnology (Shanghai, China) performed LC-MS/MS protein identification.

4. Cell transfection and lentiviral packaging

The human LUAD cell lines A549, H1299, PC9, and SPCA-1 were obtained from the American-Type Culture Collection (ATCC). These cells were maintained in RPMI 1640 medium (CellMax, China) supplemented with 10% fetal bovine serum (FBS, Sigma-Aldrich) under standard conditions at 37 °C and 5% CO2.

The MND1 plasmid, PABPC1 plasmid, and RCOR2 plasmid, along with their corresponding control vectors, were obtained from Miaoling Biotechnology (Wuhan, China). The MND1 CRISPR-Cas9 knockout vector construction was sourced from General Biosystems (http://www.generalbiol.com/). The targeted sgRNA sequence for MND1 knockout was as follows: 5′-AGAAAAGAGAACTCGCATGA-3′; 5′-CATCATCAACTAAGCTTTGA-3′; 5′-ACAAACTTACCGTTTCACAT-3′. RCOR2-shRNA and scrambled control vectors were acquired from Genechem (Shanghai, China). The target sequences are presented as follows:

shCtrl: 5′-GCAAGCTGACCCTGAAGTTCA-3′;

shRCOR2#1: 5′-GATCCGCGTTGGAACCAATTA-3′;

shRCOR2#2: 5'- GGCATGCTTCTGTGGCATAAG -3';

shRCOR2#3: 5′- CCGTAGGTATGGCAAAGACTT -3′;

shRCOR2#4: 5′- CAGCCTGGTGAAATACTACTA -3′.

All constructs were validated through DNA sequencing.

Co transfection of target plasmids and lentiviral packaging plasmids into 293T cells. After 48 hours, the viral supernatant was collected, sterilized by filtration through a 0.45 μm membrane, and subsequently utilized to infect LUAD cells in the presence of 1 μg/ml polybrene (HY-112735, MedChemExpress, America). After 48 hours of incubation, the viral solution was replaced with fresh medium supplemented with puromycin (HY-B1743, MedChemExpress, America) for selection, establishing stable cell lines.

5. mRNA stability experiment

Cells were incubated with 5µg/ml Actinomycin-D at different time points. After reaching the corresponding time points, cells were collected and total RNA was isolated. Genomic DNA was removed from the extracted RNA, and the extracted RNA was reverse transcribed into cDNA. RT-qPCR detection of mRNA expression levels of target genes and reference genes, followed by the calculation of the target RNA's half-life.

6. Immunohistochemistry (IHC) assay

Paraffin sections were deparaffinized in xylene and then rehydrated using a gradient ethanol series. Antigen retrieval was performed by microwave heating for 15 min in 1× Sodium citrate buffer, followed by a 30-min cooling period at room temperature. Endogenous peroxidase activity was then blocked by incubating the sections in 3% hydrogen peroxide solution for 10 minutes. After blocking nonspecific binding sites with 3% BSA for 1 h at room temperature, the tissue sections were incubated with appropriately diluted primary antibodies overnight at 4 °C. The following day, the sections were further incubated with biotinylated secondary antibodies (goat anti-rabbit or anti-mouse IgG) for 30 minutes at room temperature. Immunoreactive signals were then visualized using diaminobenzidine (DAB) as the chromogenic substrate, followed by counterstaining with hematoxylin to enhance tissue morphology. Antibodies against MND1 (1:200), KLF4 (1:200), SOX2 (1:200) and RCOR2 (1:200) were used.

7. Dual-luciferase reporter gene assay

Cloning the 3 '- UTR of RCOR2 into the downstream region of firefly luciferase ORF to construct a luciferase reporter gene, which is under the transcriptional control of the PGK promoter between NheI and SalI sites. Mutant reporter constructs were generated by truncating the motif (611-635) within the RCOR2 3'-UTR. The pmirGLO constructs containing either the wild-type or mutant RCOR2 3'-UTR were transiently transfected into HEK293 cells with overexpression of MND1, PABPC1, or control. At 48 hours post-transfection, cells were collected and luciferase intensity was measured. Dual-luciferase reporter gene assays were performed as described above.

8. Flow cytometric analysis

Resuspend cells in PBS supplemented with 3% BSA and incubated with CD133 antibody (#666666-1-Ig, Proteintech) and CD44 antibody (561858, [BD Pharmingen](https://www.bdbiosciences.com/zh-cn/products/reagents/flow-cytometry-reagents/research-reagents/single-color-antibodies-ruo/pe-mouse-anti-human-cd44.561858)) at 4°C for 1 hour. The labeled cells were incubated with FITC-conjugated goat anti-mouse secondary antibody for 30 min and subsequently subjected to flow cytometric analysis (BD Biosciences).

Treat lung adenocarcinoma cells with oxaliplatin and Gemcitabine for 24 hours, and collect live cells. After treating the cells with an apoptosis assay kit (40302ES50, Yeasen, China), the labeled cells were analyzed using a FACSCalibur flow cytometer (BD Biosciences).

9. Wound-Healing Assay

In a six-well plate, about 2.5×10^3^ cells were planted and cultured for twenty-four hours. Scratch perpendicularly to the six-well plate using a 200 μL pipette tip. For incubation, serum-free media was then added. Take pictures and use a microscope to record the scratch width at 0 and 24 hours. The average distance between cells and the healing rate was computed using ImageJ software.

10. Animal experiment

For animal experiments, we chose to culture a sufficient number of SPCA-1-sgCtrl/koMND1/koMND1-oeRCOR2 cell lines. For anesthesia, mice randomly assigned to the experimental group were injected intraperitoneally at a dose of 10 mg/kg sodium pentobarbital. Separate the cells into a single-cell suspensions (1×10^6^) and injected them subcutaneously into the 4-week-old female BALB/c nude mice; there were 5 mice in each group (n = 5), and the total number of experimental mice was 15. When the tumor volume reached approximately 500 mm3, the xenograft mice were sacrificed by CO_2_ inhalation, and tumor formation and tumor growth curves were observed, the mice were observed for a period of 42 days. All animal operations strictly follow guidelines of the Institutional Animal Care and Use Committee, and all protocols were approved by the Animal Ethics Committee of Hunan Normal University.

**Supplementary Figures:**

**Fig.S1 MND1 is significantly correlated with the stemness of LUAD**

(A) Differential gene expression analysis of extreme values (high vs low) of MND1 in 250 samples from TCGA-LUAD. (B, C) Construct a co-expression gene network with the optimal soft threshold set to the power of β=4. (D) The violin plot displays QC metrics of cells included in GSE119911 and GSE148071, including the percentage of mitochondrial gene counts (Percent_mt) and the number of genes (nFeature_RNA), segmented by the study. (E, F) Cell types and UMAP plot of cells in GSE119911 and GSE148071. (G) The heatmap illustrates the top ten expressed genes in different cell clusters. (H) Selected cancer cell subgroups were subjected to pseudotemporal analysis. (I) Pseudotemoral analysis reveals that the selected cancer cell subgroups can be divided into 9 states. (J) Heat map indicates gene expression trends in cell subpopulations. (K) GSEA enrichment plots of regulated RCD gene sets in 500 LUAD samples. (L) Scatter plots depicting the correlation of MND1 mRNA expression with six PANoptosis-associated genes (APBB1PIP, NLRP3, CASP9, RIPK1, RIPK3, TNFRSF1B). Statistical analyses were performed using Prism GraphPad Software and tested by Pearson’s correlation analysis.

**Fig.S2 MND1 deficiency synergistically induces PANptosis with gemcitabine treatment**

(A, B) Representative images of control and MND1-overexpressing LUAD cells treated with 10 μg/mL of oxaliplatin and gemcitabine, respectively, for 24 hours. Positive YP1 (green) indicates necrotic apoptosis or apoptotic cells; positive PI (red) indicates necrotic apoptosis, apoptosis, or pyroptosis of cells. (C, D) Representative images of control and MND1 knockout LUAD cells treated with 10 μg/mL of oxaliplatin and gemcitabine, respectively, for 24 hours. Positive YP1 (green) indicates necrotic apoptosis or apoptotic cells; positive PI (red) indicates necrotic apoptosis, apoptosis, or pyroptosis of cells.

**Fig.S3 MND1 promotes the stemness of LUAD cells**

(A) RT-qPCR was performed to assess changes in stemness markers in cell lines with stable overexpression or knockout of MND1. (B) Flow cytometry was employed to detect the proportion of CD44+ cells in cell lines with stable overexpression or knockout of MND1. Data in the graphs are presented as mean ± SD. All data in bar graphs were assessed by two-tailed Student’s t-test or one-way ANOVA with multiple comparisons. *P < 0.05, **P < 0.01, ***P < 0.001, ****P < 0.0001.

**Fig.S4 RCOR2 is a downstream target gene of MND1**

(A) Venn diagram showing genes that are downregulated in knockout MND1 cell lines and intersect with turquoise modules. (B) The correlation network diagram shows the correlation between intersection genes and mRNAsi. (C) RT-qPCR showed the expression of RCOR2, RHEBL1, NEIL3, SAP30 and TEX19 in MND1 knockout or overexpression cell lines. (D) Western blotting analysis detected RCOR2 in MND1-overexpressing and depletion cells. Data in the graphs are presented as mean ± SD. All data in bar graphs were assessed by two-tailed Student’s t-test or one-way ANOVA with multiple comparisons. ns nonsignificant (P >0.05), *P < 0.05, **P < 0.01, ****P < 0.0001

**Fig.S5**

(A) Representative images of tumor spheres formed by MND1 knockout and RCOR2 overexpressing cells. (B) Representative images of tumor spheres formed by MND1 overexpressing and RCOR2 knockdown cells. (C) Semi-quantitative analysis reveals a positive correlation between MND1, RCOR2, and KLF4/SOX2. (D) Western blot analysis was employed to measure the expression levels of PANoptosis-related markers in RCOR2 knockdown MND1 overexpression cells and RCOR2 overexpression MND1 knockout cells treated with gemcitabine for 24 hours. Statistical analyses were performed using Prism GraphPad Software and tested by Pearson’s correlation analysis.

**Fig.S6 MND1 forms an interaction with PABPC1**

(A) Enriched clustering of proteins interacting with MND1 by GO biological processes and KEGG pathways. (B) Interaction network diagram of the top 50 proteins interacting with MND1 by mass spectrometry analysis. (C) Actinomycin D (10 μg/mL) was applied to LUAD cells with combined intervention of MND1 and PABPC1. RNA was extracted at designated time points to detect RCOR2 mRNA levels. (D, E) RIP assay in H1299 cells showed that inhibition of PABPC1 inhibits MND1 and PABPC1 binding to RCOR2 mRNA; anti-IgG was employed as a negative control. (F, G) RIP assay in SPCA-1 cells shows that complementation of PABPC1 increase MND1 and PABPC1 binding to RCOR2 mRNA; anti-IgG was employed as a negative control. (H) PABPC1-binding motif predicted by bioinformatics analysis. (I) The RCOR2 3'UTR region is shown in the purple box. The orange box represents the potential PABPC1 binding site (WT, upper panel), and the lower panel shows the RCOR2 3'UTR region with the PABPC1 binding site truncated. (J) PABPC1 and MND1 overexpression enhanced luciferase activity in HEK293 cells transfected with the RCOR2 3'UTR wild-type reporter construct compared with cells transfected with the mutated reporter construct. Data in the graphs are presented as mean ± SD. All data in bar graphs were assessed by two-tailed Student’s t-test or one-way ANOVA with multiple comparisons. ns nonsignificant (P >0.05), **P < 0.01, ****P < 0.0001.

**Supplementary table：**

**Table S1 Primers for qPCR.**

| Primer | Forward Sequence | Reverse Sequence |
| --- | --- | --- |
| MND1 | 5′-CTTCGAGACCAAAGGGAACAGC-3′ | 5′-TCAGTCCATCTGTTAGCAGCTTC-3′ |
| RCOR2 | 5′-TCCGAGGTCTTGACTCTCAGCT-3′ | 5′-CGGGAGTTGAACTTGGTGTTGG-3′ |
| KLF4 | 5′-CATCTCAAGGCACACCTGCGAA-3′ | 5′-TCGGTCGCATTTTTGGCACTGG -3′ |
| SOX2 | 5′-GCTACAGCATGATGCAGGACCA-3′ | 5′-TCTGCGAGCTGGTCATGGAGTT-3′ |
| ABCC1 | 5′-CCGTGTACTCCAACGCTGACAT-3′ | 5′-ATGCTGTGCGTGACCAAGATCC-3′ |
| MYC | 5′-CCTGGTGCTCCATGAGGAGAC-3′ | 5′-CAGACTCTGACCTTTTGCCAGG-3′ |
| EPCAM | 5′-GCCAGTGTACTTCAGTTGGTGC-3′ | 5′-CCCTTCAGGTTTTGCTCTTCTCC-3′ |
| β-actin | 5′-CACCATTGGCAATGAGCGGTTC-3′ | 5′-AGGTCTTTGCGGATGTCCACGT -3′ |

**Table S2**

**TP53 Status of LUAD Cell Lines Used in This Study**

| Cell line | Tp53 status |
| --- | --- |
| A549 | Wild-type |
| H1299 | Null |
| PC9 | Mutant |
